# The role of mitochondria-associated membranes mediated ROS on NLRP3 inflammasome in cardiovascular diseases

**DOI:** 10.3389/fcvm.2022.1059576

**Published:** 2022-12-14

**Authors:** Jiahao Zhao, Junli Li, Guoyong Li, Mao Chen

**Affiliations:** ^1^Laboratory of Heart Valve Disease, West China Hospital, Sichuan University, Chengdu, China; ^2^Regenerative Medicine Research Center, West China Hospital, Sichuan University, Chengdu, China; ^3^Department of Cardiology, West China Hospital, Sichuan University, Chengdu, China

**Keywords:** reactive oxygen species, mitochondria-associated membranes, NLRP3 inflammasome, myocardial infarction, myocardial hypertrophy, atherosclerosis

## Abstract

Reactive oxygen species (ROS) metabolism is essential for the homeostasis of cells. Appropriate production of ROS is an important signaling molecule, but excessive ROS production can damage cells. ROS and ROS-associated proteins can act as damage associated molecular pattern molecules (DAMPs) to activate the NACHT, LRR, and PYD domains-containing protein 3 (NLRP3) inflammasome in cardiovascular diseases. Previous studies have shown that there are connected sites, termed mitochondria-associated membranes (MAMs), between mitochondria and the endoplasmic reticulum. In cardiovascular disease progression, MAMs play multiple roles, the most important of which is the ability to mediate ROS generation, which further activates the NLPR3 inflammasome, exacerbating the progression of disease. In this review, the following topics will be covered: 1. Molecular structures on MAMs that can mediate ROS generation; 2. Specific mechanisms of molecule-mediated ROS generation and the molecules' roles in cardiovascular disease, 3. The effects of MAMs-mediated ROS on the NLRP3 inflammasome in cardiovascular disease. The purpose of this review is to provide a basis for subsequent clinical treatment development.

## Introduction

Mitochondria are essential for maintaining cellular homeostasis and are particularly important for cells with high energy consumption, such as continually contracting cardiac cells (1). Enhanced reactive oxygen species (ROS) in cells are caused by mitochondrial dysfunction and endoplasmic reticulum (ER) stress driven by intracellular or extracellular stimulation (2, 3). In cardiovascular disorders, abnormal mitochondrial ROS metabolism increases mitochondrial dysfunction and inflammation, causing inadequate energy supply, oxidative stress, and even cell death, speeding the disease's destructive course (4–7).

Mitochondria are not considered independent and static structures because they communicate intimately with other organelles, including the ER, cytoskeleton, and nucleus (8). Mitochondria-associated membranes (MAMs), dynamic platforms in mitochondria and ER contact sites, are specific microdomains to sort vital and dangerous signals and regulate signal transduction pathways (9). Notably, a large number of regulatory proteins reside at MAMs, resulting in a structural bridge for the functional clustering of molecules (10–12); thus, Ca^2+^ homeostasis, lipid metabolism, and ROS production are all regulated at MAMs (13, 14). Because of their essential role in cells, the dysfunction of MAMs has the potential to determine the fate of cells. For instance, knocking down Ca^2+^ transport complexes at MAMs reduces cytoplasmic and mitochondrial Ca^2+^ levels and contributes to mitochondrial dysfunction and heart failure (15). Dysfunctional MAMs trigger excessive ROS, including mitochondria ROS (mtROS), which result in severe oxidative damage of intracellular biomacromolecules, ultimately promoting chronic cardiovascular disease (16). Knockout of caveolin-1, a lipid metabolism regulator at MAMs, aggravates cardiac dysfunction after myocardial ischemia by disturbing lipid metabolism (17). In addition, recent studies have shown that the formation of inflammasomes is also closely related to MAMs. Therefore, MAMs have recently received increasing attention and have been proposed as a new promising therapeutic strategy for various diseases, including cardiovascular disease.

The NACHT, LRR, and PYD domains-containing protein 3 (NLRP3) inflammasome is a multiprotein complex activated by MAM-derived effectors and recruited to form at MAMs (18, 19). The NLRP3 inflammasome initiates innate immune responses by sensing various danger signals (20) and plays a crucial role in the pathological progression of atherosclerotic vascular injury, ischemic heart disease, and non-ischemic heart disease that cause cardiac dysfunction (21–23). Despite this association, the relationship between ROS and NLRP3 inflammasome at MAMs remains unclear.

## Production of ROS at MAMs

### Sources of ROS

ROS refer to free radicals and non-free radical forms of oxygen, including superoxide anion (O^2−^), hydrogen peroxide (H_2_O_2_), hydroxyl radical (OH^−^), ozone (O_3_), and singlet oxygen (O_2_) (24). ROS participate in physiological reactions as redox messengers during processes of cell growth, differentiation, and angiogenesis (25). Moderate ROS-mediated actions assist cells in re-establishing the redox balance (i.e., redox homeostasis) and then protect cells against ROS-induced oxidative stress. However, the imbalance of ROS generation and elimination induces oxidative stress, contributing to several cardiovascular pathological progression, such as atherosclerosis, diabetic cardiomyopathy, and hypertension (26). ROS can be produced by the mitochondria (27), the ER (28), and the cytoplasm and the mitochondria are the most important organelle for ROS production.

#### ROS derived from electron transport chain (ETC)

The ETC of mitochondrial oxidative phosphorylation (OXPHOS) is made of enzymatic complexes (I-V), including four multimeric enzymes, two mobile electron carriers, and an adenosine triphosphate (ATP) synthase. During the process of OXPHOS, molecular oxygen receives electrons at complex IV and is ultimately reduced to H_2_O. During the process of electron transport in the ETC, they pass through complex I and complex III first, causing O_2_ to form superoxide (29).

#### ROS derived from mitochondrial membrane and matrix

Dehydrogenases such as pyruvate dehydrogenase and ketoglutarate dehydrogenase reside in the mitochondrial matrix. The mitochondrial outer membrane houses cytochrome b5 reductase and monoamine oxidases. Mitochondrial inner membrane enzymes include glyceraldehyde-3-phosphate dehydrogenase, dihydroorotate dehydrogenase, and electron transfer flavin protein-ubiquitin oxidoreductase. When these enzymes perform their cellular function, they may generate ROS (30).

#### ROS derived from proton-motive force (PMF)

During electron transfer in the ETC, the released energy can cause complexes I, III, and IV to transport protons from the mitochondrial matrix across the membrane to the intermembrane space in reverse concentration. The electrochemical H^+^ gradient is generated on both sides of the inner membrane of mitochondria, resulting in PMF. As electrons are transferred through the ETC, PMF markedly increases, as well as mtROS production (24). There are few studies on ER ROS because of limited testing tools. The main sources of ER ROS are cytochrome P450 family members (31) and NADPH oxidase 4 (Nox4) (32), which is also reported to be located in the mitochondria.

### Molecules mediated-ROS production at MAMs and its role in cardiovascular diseases

**Figure 1:**
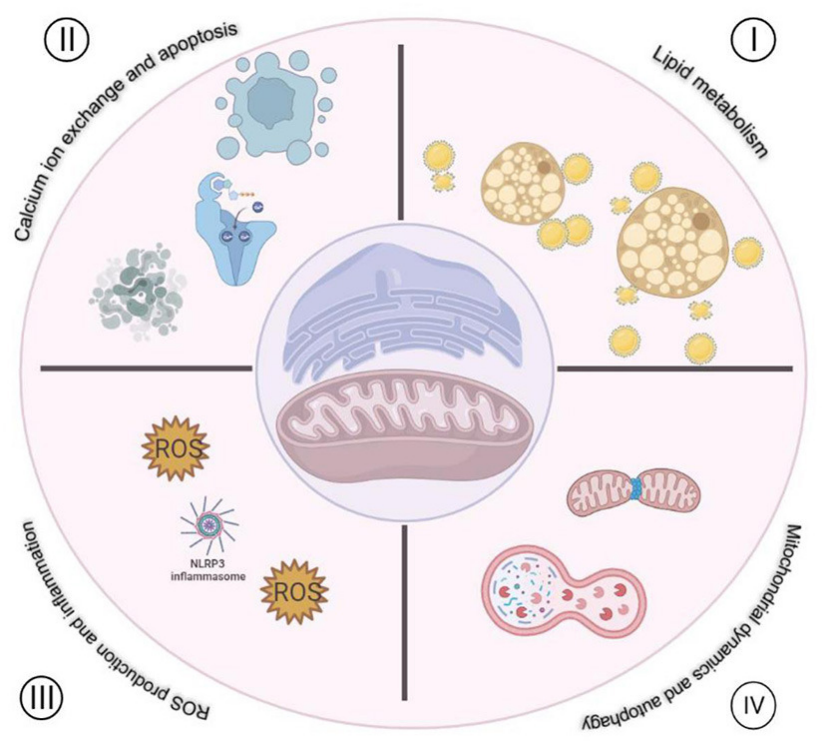
Functions of MAMs in cardiovascular disease. **I**. Regulation of lipid metabolism. **II**. Regulation of Ca^2+^ exchange between mitochondria and ER. **III**. Regulation of ROS production and inflammation. **IV**. Regulation mitochondria dynamics and autophagy.

**Figure 2:**
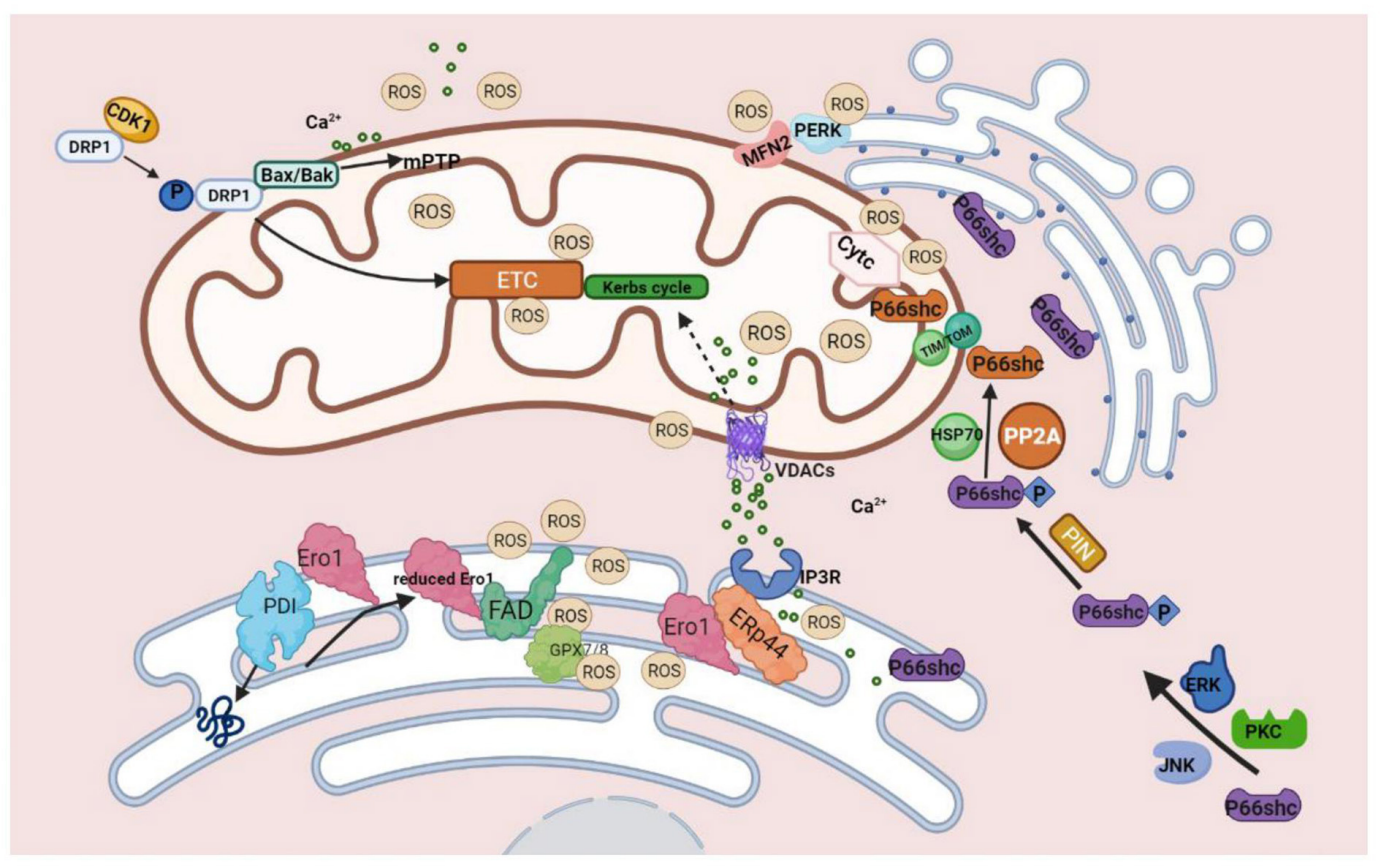
Molecules on MAMs mediated ROS production. During the process of protein folding, PDI catalyzes the formation of disulfide bonds, and it is reduced in this process. The reduced PDI is then oxidized and regenerated by ERO1 and participates in the cycle of the catalytic reaction. At this time, the reduced ERO1 transfers electrons to molecules of oxygen with the assistance of the coenzyme FAD and finally releases ROS. The generation of ROS by ERO1α does not accumulate in or leak from the ER because of GPx7, GPx8, which can eliminate excessive ROS. During ER stress, ERO1α oxidizes IP3R1, causing the detachment of ERP44, which is found in MAMs and can bind to IP3R1 to inhibit the transfer of Ca^2+^ to mitochondria from IP3R1 to promote the release of Ca^2+^ from ER, ultimately leading to excessive mtROS production finally.P66SHC is phosphorylated with the assistance of ERK, JNK, and PKC kinases. Ser36 of the CH2 domain forms Ser-Pro bone under the influence of Pin1, and then P66shc is transported into the mitochondria via TOM/TIM with the assistance of dephosphatase PP2A and chaperone HSP70. P66SHC binds to oxidized cytochrome c to generate ROS. Three pathways of DRP1-mediated ROS production: 1. Under the influence of CDK1, phosphorylated DRP1 translocates to mitochondria and impairs ETC, leading to mitochondrial dysfunction and increased production of ROS. 2. Drp1 binds to BAX/BAK directly, leading to the formation of mPTP, which can produce excessive ROS. 3. Ca^2+^ promotes the translocation of DRP1 and participates in mPTP formation, which eventually leads to mitochondrial dysfunction and increases ROS production. MFN2 can interact with PERK to reduce ROS production.

As mentioned above, MAMs play multiple biological functions in cells (Figure 1), one of which is to regulate ROS metabolism. Molecules that are localized on MAMs, including endoplasmic reticulum oxidoreductase 1 (ERO1), P66SHC, Sigma-receptor1 (SIG-1R), mitofusin1/2 (MFN1/2), VDACs, etc. These molecules are not only involved in Ca^2+^ regulation, but also participate in ROS production (Figure 2), which is crucial for cellular homeostasis. Abnormal ROS production is associated with several types of cardiovascular disorders. However, little literature summarizes the relationship between ROS and molecules located at MAMs and discusses the role of MAMs-related ROS in the development of cardiovascular diseases thoroughly. Therefore, this review focuses on the knowledge regarding ROS and cardiovascular diseases from the point of view of MAMs.

#### VDAC1

VDACs are 30 kilodalton (KD) proteins positioned on the outer mitochondrial membrane and MAMs to create a 3-nm-wide channel in all eukaryotic cells (33, 34). The three subtypes of VDACs are VDAC1, VDAC2, and VDAC3 (35, 36). And VDAC1 has been the most comprehensively investigated (37, 38). VDACs function as anchor sites for several proteins and enzymes, including hexokinases (HK) (39, 40), apoptotic proteins (Bax, Bak, and Bim), and antiapoptotic proteins (Bcl2 and Bcl-xL) (41–45). They regulate cellular energy metabolism (46), metabolic substance transport, programmed cell death (47), and ROS production.

##### The process of VDAC-mediated ROS production

Following VDAC opening, respiratory substrates flow into the mitochondria to participate in the Krebs cycle to produce reduced form of nicotinamide-adenine dinucleotid (NADH) that can enter into the ETC. The electron pair of NADH flows through the ETC to the final acceptor, O_2_. While electrons flow through the ETC, single electron leaks from complexes I, II, and III to form superoxide anion (O^2−^) (48).

##### VDAC1 in cardiovascular diseases

In a rat model of myocardial infarction (MI), VDAC1 is clearly increased, and inhibition of VDAC1 relieves excessive fibrosis in the atrial myocardium (49). One of the VDAC1 regulated mechanisms is to protect cardiac function by inhibiting the expression of VDAC1. For example, in H_2_O_2_-treated H9C2 cells, MIP2 can bind to VDAC1 and inhibit its expression, alleviating H_2_O_2_-induced cell damage (50).

Another protective mechanism targeting VDAC1 is to inhibit VDAC1 phosphorylation. VDAC1 phosphorylation is enhanced in cardiomyocytes after anoxia/reoxygenation (A/R) accompanied by increased ROS levels, collapsed mitochondrial membrane potential, increased mitochondrial permeability transition pore (mPTP) opening, and subsequent release of cytochrome c into the cytoplasm, which leads to increased apoptosis. The dephosphorylation of VDAC1 is mainly regulated by the AKT-GSK3β and p38 MAPK pathways (51, 52).

Inhibition of VDAC1 oligomerization also has protective effects on cardiac microcirculation endothelial of cells. Under ischemia-reperfusion injury (IRI), melanin restores the engagement of VDAC1 with hexokinase II (HKII) to restrict mitochondrial fission, which inhibits mPTP opening and PTEN induced kinase 1 (PINK1)/PARKIN activation, eventually blocking mitophagy-mediated cell death (53). Contradictorily, Yang et al. demonstrated that knocking out VDAC1 in H9C2 cells enhances oxidative stress-mediated cell apoptosis. The potential mechanism behind this enhanced apoptosis is that VDAC1 is directly linked to the reduction of mitochondria-bound HKII and concomitantly enhances ROS production (54). Consistent with these results, enhanced non-radical oxidant peroxynitrite (ONOO^−^), one kind of ROS that can cause mitochondria dysfunction and cell death, induces VDAC1 oligomerization in the IRI-damaged heart, which may decrease VDAC1 binding with HKII. Additionally, researchers have found that production of ONOO^−^ during cardiac IRI induces tyrosine nitration of VDAC1, delays repolarization of membrane potential, and also leads to cytochrome c release from mitochondria (55).

Inflammation plays an important role in the progression of atherosclerosis, and evidence has shown that VDAC1 may have a key function in this progression. VDAC1 protein levels are increased in THP-1 macrophages induced by external stimulation. When VDAC1 is inhibited by drugs, the production of ROS and apoptosis-related biochemical changes can be significantly inhibited. Specific siRNA can down-regulate the expression of VDAC1 and abolish cell apoptosis (56).

#### ERO1

In mammals, ERO1 has two isoforms, ERO1α, and ERO1β (57–59). Both isoforms serve similar functions. ERO1β's involvement in MAM-mediated ROS generation is insufficiently described. Hence it is not discussed in this review.

ERO1α, expressed by the *ero1a* gene, is activated by hypoxia-inducible factor 1, as shown in a rat model of ischemic stroke (60, 61). Approximately 75% of ERO1α is localized on the MAMs close to the ER side under physiological conditions, and during hypoxia, ERO1α is located on MAMs completely (62). ERO1α contains two key helical globular folds that possess CXXCXXC active sites, in addition to Asp 280 and Asp 384 N-glycosylation sites (63). The activity of ERO1 is regulated by a disulfide bond formed by its own four cysteines. The active ERO1 contains a Cys94-Cys99 disulfide bond while inactive ERO1 forms two disulfide bonds, Cys94-Cys131 and Cys99-Cys104, with the cysteines of other proteins, and the transformation of the structure is regulated by protein disulfide isomerase (PDI) (64).

##### The process of ERO1-mediated ROS production

ERO1 is a crucial component of the oxidative folding mechanism in ER. The folding of proteins in ER requires the participation of ERO1 and PDI. PDI catalyzes the formation of disulfide bonds during the folding of proteins and is reduced during this process. The reduced PDI is then oxidized and regenerated by ERO1 and participates in the catalytic reaction cycle. At this time, the reduced ERO1 transfers electrons to molecules oxygen by the assistant coenzyme FAD and ultimately releases H_2_O_2_. Generation of ROS by ERO1α does not accumulate in or leak from the ER because of glutathione peroxidase 7 (GPX7), and GPX8 (largely localized on MAM), which can eliminate excessive ROS.

The mtROS are generated when MAMs transfer large amounts of Ca^2+^ to mitochondria, while ERO1α plays a key role in regulating Ca^2+^ release through MAMs and inositol 1,4,5-trisphosphate receptor type 1 (IP3R1). During ER stress, ERO1 oxidizes IP3R1, causing the detachment of endoplasmic reticulum protein 44 (ERP44), which is located in MAMs and binds to IP3R1 to block the transport of Ca^2+^ to mitochondria from IP3R1 in order to accelerate the release of Ca^2+^ from PDI, ultimately resulting in excessive mtROS generation. ERO1α can interact with IP3R1 to promote NOX2-dependent ROS generation through CAMKII by Ca^2+^ effluxed from the ER.

##### ERO1 in cardiovascular diseases

ERO1 can alter the activities of cardiomyocytes due to its capacity to regulate Ca^2+^ signaling and ROS production. Transverse aortic constriction (TAC) surgery in wild-type (WT) mice and ERO1α ablation mice confirmed that deletion of ERO1α protects mice from progressive heart failure. At the mechanic level, ERO1α expression under pathological conditions disturbs the redox state of MAMs, causing an imbalance in Ca^2+^ homeostasis and ultimately leading to myocardial disease (65).

ERO1α is implicated in arrhythmias in hypertrophic cardiomyopathy. A previous study has reported that inhibition of ERO1 has treatment potential in hypertrophic hearts since it stabilizes the RyR2-ERp44 complex, lowering spontaneous Ca^2+^ release, and Ca^2+^-dependent tachyarrhythmias without generating hypoxia-induced oxidative stress in the sarcoplasmic reticulum (SR) (66).

The widely-used *apoe*^−/−^ atherosclerotic mouse model has found that increased macrophage apoptosis is accompanied by increased ERO1α expression. Inhibition of ERO1α revealed decreased macrophage apoptosis and smaller atherosclerotic plaques, with similar results *in vitro*. To date, no specific mechanism for these effects has been elucidated (67).

ERO1 exerts a negative function in cardiovascular disease. In the absence of ERO1, cardiovascular disease development is mitigated, but its overexpression accelerates pathogenic processes.

#### P66SHC

The shc family has three members that encode 46KD, 52KD, and 66KD proteins, all signaling adaptor proteins in cells. They are known as P46SHC, P52SHC, and P66SHC and are located at the same genetic locus of the chromosome (68). P66SHC comprises 583 amino acid residues, with a 146 amino acid CH2 domain at the N-terminal end. Following the CH2 domain is the PTB domain consisting of 195 amino acid residues, containing three α helices and seven β-sheets, which can bind to cytochrome c (69). Unlike other shc family members, P66SHC distributes in the heart, liver, and lung and expresses in all celluar types except hematopoietic stem cells (70). It is located on the MAMs, mitochondria, and cytoplasm (71).

##### The process of P66SHC-mediated ROS production

In physiological conditions, P66SHC acts in an inactive, oligomeric state. However, P66SHC becomes tetrameric and exerts biological activity in response to oxidative stress (72). The most critical function of P66SHC is regulating intracellular oxidative stress and ROS production in cells (29, 70, 73, 74). In response to oxidative stress stimulating factor (UV, H_2_O_2_), Ser36 on P66SHC is phosphorylated by the assistance of P38 MAPK, ERK, JNK, and PKC kinases (75–77). In addition to Ser36 being phosphorylated, Ser54 and Thr386 are also phosphorylated for maintaining the stability of their structure and preventing P66SHC from being degraded by ubiquitination (78). In oxidative stress, P66SHC translocates to mitochondria from MAMs fraction. Ser36 of the CH2 domain forms Ser-Pro bone under the influence of prolylisomerase Pin1, then P66SHC is transported into mitochondria *via* TOM/TIM with the assistance of dephosphatase PP2A and chaperone HSP70. The PTB domain of P66SHC binds to oxidized cytochrome c, then from which electrons transfer to molecular oxygen to generate ROS.

##### P66SHC in cardiovascular diseases

Compared with WT mice, *p66shc* knockout mice have no changes in heart rate and blood pressure, but an increase in the number of cardiomyocytes in physiological conditions (79). One possible reason is that P66SHC facilitates the apoptosis of cardiomyocytes.

P66SHC plays a protective role in short-term ischaemia. Following IRI, *p*66*shc*^−/−^ and transiently silenced *p66shc* mice have increased infarct size compared with WT mice. Mechanically, depletion of *p66shc* is associated with a reduction in phosphorylation levels of AKT at Thr308 and STAT3 at Ser727 and causes mitochondrial swelling and cell apoptosis *via* CASPASE3 (80). In contrast to the results above, the absence of *p66shc* reduces oxidative stress and release of lactate dehydrogenase from the coronary outflow and exerts a protective effect in isolated perfused hearts after IRI (81).

In mice with coronary artery ligation for 7 days, P66SHC expression increases in one week. *P66shc* knockout (KO) mice have improved survival and reduced rate of cardiac rupture post-MI compared with WT mice. In KO mice subjected to MI, expression of matrix metalloproteinase 2 (MMP2) in hearts is reduced, fibroblast activation and collagen accumulation are promoted, and oxidative stress is attenuated, leading to reduced reactive fibrosis and left ventricular dilation. These results collectively suggest P66SHC is involved in adverse cardiac remodeling after MI (82).

In mice with TAC, the absence of *p66shc* enhances the expression of phosphodiesterase type 5 (PDE5) to reduce oxidative stress damage, thereby reducing cardiac dysfunction (83). AngiotensinII (Ang II) induces left ventricular hypertrophy and apoptosis of cardiomyocytes and endothelial cells in WT mice. However, *p*66*shc*^−/−^ mice can inhibit the pro-apoptotic/hypertrophic effect of AngII, consistent with cardiomyocyte experiments *in vitro*. These results indicate that P66SHC plays a protective role in hypertrophic cardiac injury induced by AngII (79).

P66SHC-Ser36 is phosphorylated in both cardiomyocytes and cardiac fibroblasts via a mitogen-activated protein kinase (MEK)-dependent mechanism under the influence of thrombin, which is involved in signaling pathways that affect cardiomyocyte growth and survival and extracellular matrix remodeling. Pasteurella multocida toxin (PMT), a Galpha (q) agonist that promotes cardiomyocyte hypertrophy, induces P66SHC expression through mechanisms of protein kinase C (PKC) and MEK activity. These results indicate that P66SHC is a hypertrophy-inducing mediator of cardiomyocyte apoptosis and heart failure (84).

Interestingly, another article shows that alpha (1)-Ars require the participation of epidermal growth factor receptor (EGFR) and PKC epsilon to promote phosphorylation of P66SHC-YY (239/240) and P66SHC-S (35) and further activate the AKT signaling pathway. AKT pathways selectively phosphorylate/inactivate forkhead box o (FOXO) transcription factors to down-regulate downstream gene expression to reduce cell damage. Silencing P66SHC by siRNA results in reduced expression of FOXO3A-regulated genes such as *mnsod, p27kip1*, and *bim-1* as well as cardiomyocyte hypertrophy, suggesting that P66SHC has an anti-hypertrophic effect in cardiomyocytes (85).

Long-term treatment of WT mice with a 21% high-fat diet increases the cumulative early lesion area of the aorta by around 21%, but only by 3% in *p*66*shc*^−/−^ mice. These studies also show that *p*66*shc*^−/−^ mice have fewer macrophage-derived foam cells and apoptotic vascular cells in early lesions compared with WT mice. In addition, systemic and tissue oxidative stress is significantly reduced in *p*66*shc*^−/−^ mice but not in WT mice. These findings support the notion that *p*66*shc*^−/−^ mice may play a crucial role in regulating systemic oxidative stress and vascular disease. These results suggest that P66SHC may serve as a molecular target for the therapy of vascular disorders (86). In recent research that evaluated P66SHC mRNA in peripheral leukocytes (WBC) and subcutaneous fat samples from individuals with high and low density lipoprotein (LDL) plasma levels, the association between lipids, oxidative stress, and P66SHC was also hypothesized. In this research, patients with high LDL have substantially higher WBC and adipose tissue P66SHC mRNA levels than those with low LDL. In addition, LDL plasma level was shown to be the sole variable associated with P66SHC mRNA expression (87).

#### MFN2

MFN1 and MFN2 are GTPases located in the outer membrane of mitochondria (OMM); MFN1 consists of 741 amino acids, whereas MFN2 consists of 752 amino acids (88). They are 63% homologous, but MFN2 has an additional region of the N-terminus that can bind to RAS, suggesting functional differences between MFN1 and MFN2 (89). The activity of MFN2 is regulated by SMAD2, which can act as a scaffold platform for RNI1 and MFN2, prompting MFN2 to exert GTPase activity (90). The main role of MFN1 and MFN2 is to promote mitochondrial fusion (91), whereas dynamin-related protein 1 (DRP1), Fission 1 (FIS1), and other molecules (92, 93) can cause mitochondria to divide, and all of these molecules regulate the morphological structure of MAMs and mitochondria together.

##### The process of MFN2-mediated ROS production

To date, there are few reports on the relationship between MFN1 and ROS, but MFN2 is abundant. The detailed mechanism between MFN2 and ROS production is not clearly elaborated; however, many studies have shown that MFN2 can inhibit the production of ROS, and one of the possible mechanisms underlying this production is that MFN2 can interact with PRKR-like endoplasmic reticulum kinase (PERK) to inhibit it's activity, which in turn reduces ROS production by endoplasmic stress. When MFN2 is absent, PERK activity increases, promoting the production of ROS. ROS production is reduced after overexpression of MFN2 in other studies (94–96); however, more work is needed to fully understand this relationship.

##### MFN2 in cardiovascular diseases

In both models of TAC-induced cardiac hypertrophy of mice and TGFβ-induced cardiomyogenic fiber activation assay *in vitro*, the expression of MFN2 decreased. Intracellular ROS generation decreases, and cardiac fibroblast activation is inhibited significantly when MFN2 expression is enhanced. The mechanism for the protective role of MFN2 is that MFN2 inhibits the signaling pathway of PERK/ recombinant activating transcription factor 4 (ATF4) and ROS generation (97). Another study has shown that MFN2 facilitates PARKIN translocation and phosphorylation, causing mitophagy to remove damaged mitochondria (98). In addition, another function of MFN2 is to participate in mitochondrial fusion to maintain mitochondrial quality, and knockdown of MFN2 can aggravate AngII-induced damage in cardiomyocytes (99). These results indicate that MFN2 plays a protective role in the pathological process of cardiac hypertrophy.

MFN2 plays opposite roles in MI and myocardial hypoxia-reoxygenation injury. After three days of MI, MFN2 ablation mice have more severe mitochondrial damage compared with WT mice. The MFN2^−/−^ mice produce more ROS, and enhancing the expression of MFN2 can rescue this pathological process. Compared with WT mice, myocardial infarct size was reduced by 46%, and MAM formation and ROS generation were reduced in MFN2 ablation mice in the IRI model. The main reason is that the knockdown of MFN2 leads to reduced junctions and reduced ROS production in mitochondria and the ER (100).

MFN2 also plays a protective role in atherosclerosis. It has been well-documented that overexpression of MFN2 inhibits the secretion of inflammatory factors in RAW 264.7 macrophage cells and attenuates atherosclerosis progression (101). Another study has suggested that overexpression of MFN2 can reduce plaque formation and endothelial cell injury in rabbit models of atherosclerosis (102).

#### DRP1

DRP1 anchors to OMM, and its receptor mitochondrial fission factor (MFF) together regulates mitochondrial fission (103–106). In addition, DRP1 is implicated in various membrane structures in cells, including peroxisomes, lysosomes, and plasma membranes (107, 108). Because of GTPase activity, DRP1 has membrane remodeling activity after binding to membrane structures such as the OMM or the peroxisomal membrane (109, 110). The domain of DRP1 includes the GTPase C end domain (GED), GTPase N end domain, variable domain (VD, also known as B-insert), and intermediate domain (111, 112).

##### The process of DRP1—mediated ROS production

There are three pathways for DRP1-mediated ROS production: 1. Under the influence of CDK1, S616 of DRP1 is phosphorylated, causing DRP1 translocation to mitochondria. DRP1 facilitates mitochondrial division and impairs ETC leading to mitochondrial dysfunction and increased production of ROS. 2. DRP1 binds to BAX/ BAK directly, leading to the formation of mPTP, which can produce excessive ROS. ROS, in turn, enhance the opening of mPTP, forming a vicious cycle of the procedure. 3. Ca^2+^ can not only inhibit ROS generation but also promote translocation of DRP1 and participate in mPTP formation, which eventually leads to mitochondrial dysfunction and increases ROS production (113).

##### DRP1 in cardiovascular diseases

DRP1 plays an important role in cellular hypoxia-reoxygenation injury because DRP1-mediated mitochondrial fission is a trigger for apoptosis. Under hypoxia-reoxygenation conditions, DRP1 is hyperactivated, leading to elevated permeability of MOM, Ca^2+^ overload, and increased ROS production. Subsequent mitochondrial depolarization and cytochrome c release lead to cell apoptosis. When the activity of DRP1 is inhibited, mitochondrial function is normalized and hypoxia-reoxygenation damage is reduced (114).

Numerous studies have shown that DRP1-mediated mitochondrial fission leads to mitochondrial dysfunction and cardiomyocyte death after MI. Mdivi-1, or P110, which can block DRP1-FIS1 interaction, can prevent the opening of mitochondrial transition pores and reduce infarct size.

Ca^2+^ is a key regulator of cardiac relaxation, and abnormal Ca^2+^ concentrations can cause impaired cardiac function, ultimately leading to cell death. Inhibition of DRP1 by siRNA can reduce cytosolic Ca^2+^ levels, and play a protective role in cardiovascular disease (115–117).

In phenylephrine (PE)-treated cardiomyocytes, mitochondrial volume is reduced. Suppression of DRP-1 expression by Mdivi-1 can inhibit cardiac hypertrophy. The possible mechanism is that Mdivi-1 can inhibit ROS production and further inhibit the activity of Ca^2+^/CaMKII (118).

Another study has shown abnormal morphology and dysfunction of mitochondria and increased orai1-mediated Ca^2+^ influx in a model of high glucose (HG)-induced cardiomyocyte hypertrophy. Mdivi-1 prevents HG-induced cardiomyocyte hypertrophy by decreasing DRP1 phosphorylation at S616 and increasing phosphorylation at S637. Knockdown of orai1 or inhibition of orai1 activity inhibits not only DRP1 activity, CnA, and p-ERK1/2 expression, but also alleviates HG-induced mitochondrial dysfunction and cardiomyocyte hypertrophy. However, cyclosporinA (a CnA inhibitor) and U0126 (a p-ERK1/2 inhibitor) improve HG-induced cardiomyocyte hypertrophy by promoting and inhibiting the phosphorylation of DRP1 at S637 and S616 sites, respectively. DRP1 is a downstream target of orai1-mediated Ca^2+^ influx and is activated by p-ERK1/2-mediated phosphorylation at S616 or CnA -mediated dephosphorylation at S637 (115).

Pretreatment with hydralazine decreases infarct size of MI in isolated perfused mouse hearts suffering acute IRI, and injection of hydralazine after reperfusion reduces the size of MI in mouse hearts undergoing IRI *in vivo*. Hydrazidazine's therapeutic effects may be mediated through binding to DRP1 to suppress its GTPase activity and prevention of mitochondrial fission (119).

#### SIG-1R

SIG-1R is a transmembrane protein composed of 223 amino acids, with well-studied crystal and three-dimensional structures (120, 121). SIG-1R, expressed in the heart, kidney, and brain, mainly localizes at MAMs and regulates signaling transduction and material exchange between the ER and mitochondria (122–124). In physiological conditions, SIG-1R binds to binding immunoglobulin protein (BiP)/glucose-regulated protein 78 (GRP78), whereas SIG-1R is released in pathological conditions. Numerous data have shown that SIG-1R is involved in various processes, such as ROS scavenging and Ca^2+^ homeostasis.

SIG-1R can scavenge ROS produced by mitochondrial dysfunction or regulate downstream signaling pathways caused by ROS. Many studies have reported that agonists of SIG-1R alleviate or rescue the damage to cells and tissues caused by ROS (125–127). In cardiovascular disease, fluvoxamine alleviates heart dysfunction caused by upregulating SIG-1R, which activates the AKT-eNOS signaling pathway in mice with cardiac hypertrophy (128). Activation of SIG-1R may reduce myocardium fibrosis and enhance cardiac function in a rat model of MI (129).

## The role of MAMs-associated ROS in NLRP3 inflammasome activation

### NLRP3 inflammasome

The NLRP3 inflammasome is a macromolecular protein complex composed of NLRP3, adaptor apoptosis associated speck-like protein (ASC), and the protease CASPASE-1.

NLRP3 is composed of the N-terminal pyrin domain (PYD), the C-terminal leucine-rich repeat domain (LRR domain), and a central NACHT domain, which contains ATPase activity and binds to ATP to activate NLRP3.

ASC has caspase recruitment domain (CARD) and PYD domains, which can promote ASC's interaction with NLRP3 to form a protein complex termed pyroptosome, or ASC specks (130–132). At the N-terminal, CARD of CASPASE-1 interacts with ASC, then undergoes self-cleavage to release p10, which is proteolytically active (133).

GSDMD plays a vital role in a programmed cell death called pyroptosis. The N-terminus of GSDMD has a cell death domain that is released by the catalysis of CASPASE-1 and binds to the cell membrane to form a 10–14 nm pore, prompting IL-18 and IL-1β and high-mobility group box 1 (HMGB1) secretion into the extracellular space (134, 135).

**Figure 3:**
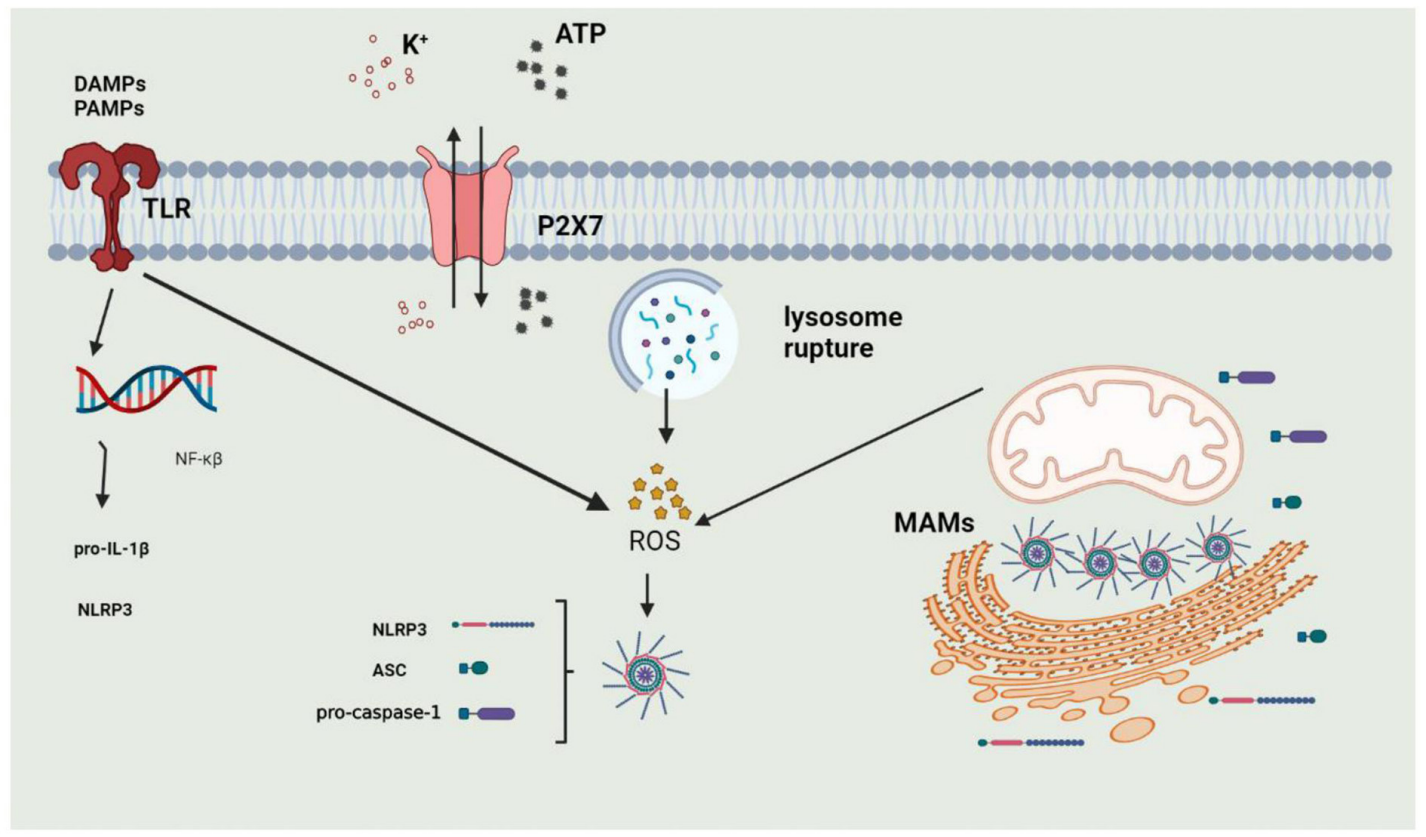
The process of activation of NLRP3 Inflammasome. 1. Priming: when DAMPs or PAMPs are recognized by TLRs or under stimulation of extracellular ATP, intracellular NF-κβ enters into the nucleus to induce the expression of NLRP3 and Pro-IL-1β; 2. activation: NLRP3 inflammasome can be activated by potassium outflux, lysosomal rupture or mitochondrial dysfunction.

The activation of the NLRP3 inflammasome is precisely regulated and considered a two-step process of priming and activation (Figure 3). The primary function of priming is to upregulate the protein expression of various inflammasome components, such as NLRP3, ASC, CASPASE-1, and pro-IL-1β. When PAMPs or DAMPs are recognized, the inflammasome is primed. PAMPs include various pathogenic bacteria and viruses, and DAMPs are mediated by PRRs to cause NF-κβ activation to participate in transcriptional regulation of inflammatory molecules. The priming step is also regulated by post-transcriptional modifications of NLRP3, including phosphorylation, ubiquitination, and hematoxylin (136–138). The activation of the inflammasome is regulated by upstream signals, including K^+^, Ca^2+^, Cl^−^, mitochondrial dysfunction, and lysosome disruption.

### MAMs-associated ROS and NLRP3 inflammasome

Under physiological conditions, NLRP3 and ASC are located in the cytoplasm and ER. When stimulated by inflammasome activators, NLRP3 and ASC translocate to MAMs. One possible reason for the process is that ROS are mainly derived from mitochondria and are unstable.

ROS accumulation can produce IL-1β in cells, suggesting that ROS can activate the NLRP3 inflammasome. However, the specific mechanism is not yet clarified. The antioxidants targeting mitochondria can prevent activation of the NLRP3 inflammasome and inflammatory response, indicating that MAMs can mediate ROS production to trigger the inflammatory response and NLRP3 inflammasome activation.

#### NOX-ROS-NLRP3 inflammasome

Endoplasmic reticulum stress (ERS) can drive NOX-mediated ROS generation, which can restore ER homeostasis (26). However, if the stimulation is prolonged or not removed in time, the ERS cannot be alleviated, and ERO1 will partially trigger the overproduction of ROS (139). Excessive ROS can cause a disorder of Ca^2+^ metabolism in mitochondria and increase mitochondrial damage (140). NOX4 activates NF-κB through ROS, consequently facilitating NLRP3 activation, and causing the release of proinflammatory cytokines (141). In response to ERS, NOX2 regulates the expression of dsRNA-activated protein kinase R (PKR) (142). Autophosphorylation of PKR causes NLRP3, CASPASE-1, and ASC to reassemble, increasing inflammasome activation. It has also been shown that ROS and K^+^ efflux caused by ERS can induce IL-1β secretion in human macrophages (143).

#### Molecules associated with MAMs-ROS-NLRP3 inflammasome

Molecules on MAMs not only preserve MAMs structural integrity but also mediate ROS generation and NLRP3 inflammasome activation. In previous work, researchers have demonstrated that MFN2 can interaction with NLRP3 to promote the NLRP3 inflammasome activation and IL-1β secretion in response to the infection of RNA virus or pathogenic bacteria (144). Another study also showed that knockdown of MFN2 can significantly reduce IL-1β secretion (145). In contrast, upregulated expression of MFN2 caused by peroxisome proliferator-activated receptor gamma coactivator 1 alpha (PGC-1α) can inhibit ROS overproduction of MAMs and inhibit activation of NLRP3 inflammasome and pyroptosis (140–146). The *mfn2* ^−/−^ mice exhibit decreased mitochondrial respiration, leading to increased ROS generation and mutational mtDNA accumulation (147). Increased ROS levels and mutational mtDNA cause the release of proinflammatory cytokines, enhancing NLRP3 inflammasome recruitment.

VDACs that regulating mitochondrial ROS production and Ca^2+^ exchange are situated on MAMs and the mitochondrial outer membrane. One study demonstrated that inhibiting VDAC greatly reduces ROS production and NLRP3 inflammasome activation (148). In order to verify the hypothesis that the NLRP3 inflammasome activation is dependent on ROS produced by respiratory mitochondria, zhou et al. suppressed mitochondrial respiration by reducing VDAC expression. The experimental results indicated that the NLRP3 activator cannot induce the IL-1β generation and CASPASE-1 activation in VDAC-deficient cells. Importantly, IL-1β production is reduced when the function of VDAC is inhibited (149). Furthermore, VDAC1 oligomerization can also increase NLRP3 inflammasome activation. NLRP3 inflammasome activators can trigger VDAC oligomerization and open mPTP. Oxidized mtDNA fragments and mtDNA in the mitochondria can exit via mPTP or VDAC-dependent channels, activating the NLRP3 inflammasome (150).

P66SHC, discussed in the preceding chapter, is an oxidoreductase on MAMs. ROS generation mediated by P66SHC activates the NLRP3 inflammasome. The increased P66SHC expression is associated with NLRP3 inflammasome activation in patients with liver fibrosis. When P66SHC is knocked down in cells, both ROS generation and NLRP3 inflammasome activation are decreased. Overexpression of P66SHC activates the NLRP3 inflammasome, but treatment with mitochondrial ROS scavenger (Mito-TEMPO) inhibits the NLRP3 inflammasome (151). These findings indicate that P66SHC can activate the NLRP3 inflammasome by generating ROS in MAMs.

DRP1 is involved in the activation of the NLRP3 inflammasome. The knockdown of DRP1 in mature oligodendrocytes prevents NLRP3 inflammasome activation, but the hyperactivation of DRP1 can cause an NLRP3-related inflammatory response by decreasing hexokinase I (HKI) (152). Other researchers have noted that enhanced ERK signaling in DRP1 knockout macrophages is associated with increased NLRP3 inflammasome activation, potentially by mediating NLRP3 mitochondrial location to promote NLRP3 inflammasome assembly (153). Furthermore, DRP1 phosphorylation is implicated in the activation of the NLRP3 inflammasome. DRP1 phosphorylation can cause excessive mitochondrial fission, ROS and mtDNA release, and NLRP3 activation. Another study found that swine influenza virus (SIV) infection causes phosphorylation of DRP1 at serine 579 and mitochondrial fission in primary porcine alveolar macrophages (PAMs), leading to an increase in IL-1β production (154). In contrast, corosolic acid (CRA) stimulates the phosphorylation of DRP1 at Ser637 to suppress mitochondrial fission and NLRP3 inflammasome activation (155).

The mitochondrial pathways signaling (MAVS) protein is located in a variety of intracellular compartments, such as mitochondria, MAMs, and peroxisomes (156). And the mitochondrial adapter protein was discovered by Subramanian et al. to mediate NLRP3 relocating and binding to the mitochondria (157). MAVS oligomerization is thought to be crucial for its signal transduction, including the activation of the NLRP3 inflammasome. Furthermore, binding to NLRP3, which is regulated by MAVS C-terminal transmembrane domain, increases MAVS oligomerization and leads to CASPASE-1 activation (158). In addition, PINK can directly combine with MAVS to block its function (159). MAVS has also been shown to promote membrane permeability or K^+^ efflux, resulting in the activation of the NLRP3 inflammasome (160).

The vesicle-associated membrane protein-associated protein B (VAPB) and protein tyrosine phosphatase-interacting protein-51 (PTPIP51) are proteins on MAMs and they can bind together to stabilize the structure of MAMs. When Tar DNA-binding protein 43 (TDP-43) breaks the linkage between them, the NLRP3 inflammasome is activated. Mechanically, the destruction of MAMs structure leads to increased production of ROS and ATP, resulting in the activation of the inflammasome (161, 162).

Hepatic inflammation can mediate the overexpression of thioredoxin interacting protein (TXNIP) and increase ROS levels with subsequent NLRP3 inflammasome activation *in vivo* and *in vitro* (163). Paclitaxel, an inhibitor of cell division, can increase the expression of TXNIP and the interaction of TXNIP-NLRP3, accompanied by increased production of ROS (164). When exposed to high glucose, ROS can up-regulate the expression of TXNIP, and the use of p38 MAPK inhibitors can inhibit the expression of TXNIP, indicating that ROS can up-regulate TXNIP expression by activating the MAPK pathway (165). These observations illustrate that ROS are related to TXNIP as well as the NLRP3 inflammasome. Moreover, a study detailed the relationship between ROS, TXNIP, and the NLRP3 inflammasome. In resting cells, TXNIP is distributed in the cytoplasm and ER and is inactive because of binding to TRX. TRX dissociates from TXNIP in a ROS-dependent manner, TXNIP binds to NLRP3 to translocate to MAMs and mitochondria, the NLRP3 inflammasome is activated, and mature IL-1β production increases (166). Binding to TXNIP can trigger conformational changes in NLRP3, and knocking down TXNIP results in NLRP3 with more S-nitrosylated sites (167). TXNIP knockdown inhibits CASPASE-1 activity and reduces IL-1β release, but TRX knockdown increases inflammasomes (166, 168), suggesting that TXNIP is a link between oxidative stress and NLRP3 inflammasome (169).

Under pathological conditions, mitochondrial Ca^2+^ overload can result in mitochondrial dysfunction, including ROS overproduction and mitochondrial pore opening, which can enhance the release of mtDNA and mtROS from mitochondria, and in turn, mtROS can oxidize mtDNA (170). mtDNA, oxidized mtDNA, and mtROS all serve as DAMPs to activate the NLRP3 inflammasome. Furthermore, when treated with mitochondrial respiratory chain inhibitors, the loss of the Δ*Ψ*m and accumulation of ROS can activate NLRP3 inflammasome and enhance IL-1β release. This process, however, is suppressed by the antioxidant 4-amino-2, 4-pyrrolidine carboxylic acid (APDC) (171). Experiments *in vivo* and *in vitro* demonstrate that the mtROS inhibitor can reduce mtROS accumulation, MAVS expression, and NLRP3 inflammasome activation (172). The ability of mtROS to enhance MAVS oligomerization suggests that MAVS may be the primary sensor of mtROS. MAVS also can bind to NLRP3 and increases its oligomerization, which activates CASPASE-1 (158).

#### Mitophagy-ROS-NLRP3 inflammasome

Mitophagy, a means of cell self-protection, can remove mitochondrial NLRP3 inflammasome activators, such as mtDNA, mtROS, and cardiolipin. Adding 3-methyladenine (3MA) (mitophagy inhibitor) or suppressing autophagy regulator autophagy protein5 (ATG5) and Beclin1 in macrophages increases ROS generation and NLRP3 inflammasome activation (173). It has been reported that mitoTEMPO can inhibit PINK1/PARKIN mediated mitophagy to inhibit NLRP3 inflammasome, alleviating cell damage (174). After lipopolysaccharide (LPS) treatment, the NLRP3 inflammasome is activated and NF-κB selectively induces the expression of SQSTM1. However, SQSTM1 depletion leads to the impairment of mitophagy and further increases NLRP3 inflammasome activation. Importantly, PRKN deficiency contributes to impaired mitochondrial recruitment of SQSTM1 and activation of the NLRP3 inflammasome. Interestingly, a recent study showed PINK1^−/−^ and PRKN^−/−^ rats exhibit enhanced NLRP3 expression and IL-1β production in myocardium following acute exercise (175). Consistently, PINK1 overexpression *in vivo* attenuates liver I/R injury, reduces ROS production, alleviates NLRP3 activation, and inhibits liver inflammation (176) and Li et al. recently provided direct evidence that FUNDC1-mediated mitophagy inhibits inflammasome activation and IL-1β production (177). However, contrary to these results, other researchers showed that mitophagy can increase the production of mtROS, which further stimulate the production of abundant mtROS dependent on mitophagy, forming a vicious cycle and ultimately leading to cell death (178, 179).

The NLRP3 inflammasome can trigger mitophagy directly, which may be one way the cell exerts its protective effect. Members of the NLR family with mitochondrial targeting sequences have been reported to combine with LC3 to initiate autophagy (180, 181). NLRP3 inflammasome activators can induce PRKN-mediated mitophagy, whereas the mitophagy in turn inhibits NLRP3 inflammasome activation. However, CASPASE-1 can inhibit mitophagy and amplify mitochondrial damage through PRKN cleavage (182, 183).

### Other MAMs-associated pathways and NLRP3 inflammasome

One of the essential functions of MAMs is the regulation of Ca^2+^ signaling, and the regulation of NLRP3 inflammasome activation by Ca^2+^ mobilization has also been demonstrated. It has been reported that blocking Ca^2+^ mobilization inhibits NLRP3 inflammasome complex activation in response to ATP stimulation. The NLRP3 inflammasome activator induces Ca^2+^ influx in macrophages *via* transient receptor potentials (TRP) cation channels, such as TRPM2, TRPM7, and TRPV2 (184, 185). The NLRP3 inflammasome is activated by Ca^2+^-sensing receptors (CaSR) and GPRC group 6 member a (GPRC6A) by decreasing intracellular cAMP and increasing Ca^2+^ (186, 187). They induce Ca^2+^ release from the ER to the cytoplasm to activate the NLRP3 inflammasome by catalyzing IP3R generation by phospholipase C. But it has also been shown that Ca^2+^ signaling is insufficient to activate the NLRP3 inflammasome unless Ca^2+^ is mobilized in a manner of mitochondrial damage.

In summary, MAMs can participate in the assembly and activation of the NLRP3 inflammasome by regulating Ca^2+^ signaling.

## The role of MAMs-associated ROS in NLRP3 inflammasome activation in cardiovascular disease

### The role of MAMs-associated ROS in NLRP3 inflammasome activation in the hypoxia-reoxygenation injury of heart

There are few studies on the impact of NLRP3 inflammasome on acute MI in humans due to the limitation of samples, whereas abundant studies have been conducted on animal models. About 3 h after ligation of the left anterior descending artery (LAD) in mice, the NLRP3 inflammasome is activated, but activity is low (188, 189). In the hypoxia-reoxygenation injury model of mice, the activity of the NLRP3 inflammasome peaks at 1 day; in contrast, it peaks at 3 days in the MI model. Because of various types of cells in the heart, studies have found that endothelial cells, cardiac fibroblasts, macrophages, granulocytes, and cardiomyocytes form NLRP3 inflammasome complexes after MI. Further, endothelial cells and cardiac fibroblasts secrete large amounts of IL-1β (190, 191).

Following I/R injury, TXNIP not only directly inhibits mTOR and activates autophagy but also inhibits autophagosome scavenging through increased production of ROS, causing cardiomyocyte apoptosis and cardiac dysfunction (192). However, overexpression of TXNIPC247S can reduce ROS generation and decrease myocardial infarct size (193). Scavengers of ROS can separate TXNIP from NLRP3 and inhibit NLRP3 inflammasome activation in cardiac microvascular endothelial cells (CMECs) simulated by I/R injury (194).

The antioxidant ethyl pyruvate (EP) can reduce phosphorylation levels of p38 and MEK, thereby inhibiting ROS-mediated NLRP3 inflammasome activation and alleviating myocardial I/R injury (195). It has also been shown that ROS overproduction and up-regulation of TXNIP following MI in beagle dogs lead to activation of NLRP3 inflammasome that further impairs JAK2-STAT3 and insulin signaling pathways, leading to high expression of PPAR-α to lipid metabolism disorder (196). It has also been found in cardiomyocytes of neonatal rats that mitochondrial fission causes increased production of ROS and activates the NLRP3 inflammasome. Thus, proteins that control mitochondrial fission, such as DRP1and FIS1, can be used as potential targets of the drug. Under hypoxia in rat cardiomyocytes, DRP1 mediates increased mitochondrial division, causing mtDNA and mtROS release from mitochondria to activate the NLRP3 inflammasome. Pigment epithelium-derived factor (PEDF) can reduce the division of DRP1 to suppress NLRP3 inflammasome activation (197).

### The role of MAMs-associated ROS in NLRP3 inflammasome activation in atherosclerosis

As described previously in this review, TXNIP dissociates from TRX and binds to NLRP3, resulting in NLRP3 inflammasome activation, which leads to the maturation and release of IL-1β and IL-18. TRX80, a truncated form of TRX-1, can also activate the NLRP3 inflammasome and cause the production of the potent atherogenic cytokines IL-1β and IL-18 (198). Byon et al. found that *txnip /apoe* double knockout mice exhibit 49% less atherosclerotic plaque in the aortic root and 71% less abdominal aortic lesions compared with control *apoe* knockout mice (199). The findings suggest that TXNIP plays a key role in the development of oxidation, inflammation, and atherosclerosis in mice. Intervention of TXNIP expression may be a potential target for the prevention and treatment of atherosclerosis and inflammatory vascular diseases. In addition to causing oxidative damage and inflammation, TXNIP can also increase carotid intimal thickness and lead to abnormal glucose metabolism. A study in the Chinese Han population reported that TXNIP single nucleotide polymorphisms individually and cumulatively increased the risk of coronary heart disease by regulating TXNIP expression and gene-environment interactions (200).

Among the seven subtypes of NOX, NOX1, NOX2, NOX4, and NOX5 are expressed in the vasculature. NOX1 and NOX2 can induce atherosclerosis by promoting endogenous and exogenous inflammation in vascular wall cells (201). Notably, loss of NOX4 accelerates atherogenesis in a variety of mouse models of atherosclerosis (202). NOX4 is widely expressed in vascular smooth muscle cells and is essential for maintaining vascular homeostasis. Overexpression of this gene leads to increased ROS levels, senescence, and susceptibility to apoptosis, which are closely related to the severity of atherosclerosis (201). However, in response to proatherogenic ERS, it has also been reported that NOX4 can regulate the unfolded protein response and subsequent autophagy activity *in vivo*, and protect vascular endothelial function to play an atheroprotective role (26).

## Conclusion

MAMs are sites of contact between mitochondria and endoplasmic reticulum, which regulate Ca^2+^ signaling and ROS metabolism, provide anchor sites for a variety of biochemical reactions, and play an essential role in maintaining cell homeostasis. Molecules on MAMs are involved in the generation of ROS, which are important cellular signaling molecules and are toxic in cardiovascular diseases. ROS are involved in the physiological and pathological processes of a variety of cardiovascular diseases, such as MI, hypertrophic cardiomyopathy, and atherosclerosis. One of the most important functions of ROS is the regulation of inflammation and NLRP3 inflammasome activation.

Extensive progress has been made in studying the structure and function of MAMs. However, additional work is still needed to explore the specific mechanism regulating the integrity of MAMs and the functional study of proteins on MAMs. This understanding may not only help to prevent and treat cardiovascular diseases but is also crucial for other diseases. Currently, there is no specific mechanism for the regulation of ROS by MAMs, and a large number of unknowns remain to be investigated. MAMs have been isolated, and the molecular components of MAMs have been identified by mass spectrometry. It is still unknown if some of these new molecules that regulate ROS generation and mediate inflammatory responses during disease. Further, the structure of these molecules, how they change under different conditions, and how they contribute to the inflammatory processes all remain unknown. Elucidating these unknowns may lay the foundation for development of new therapeutics to target cardiovascular disease.

## Author contributions

Conception and design by MC and JZ. Administrative support by JL and GL. Final approval of manuscript by all authors. All authors contributed to the article and approved the submitted version.
